# Exploring the prevalence, impact and experience of cardiac cachexia in patients with advanced heart failure and their caregivers: A sequential phased study

**DOI:** 10.1177/02692163221101748

**Published:** 2022-06-21

**Authors:** Matthew A Carson, Joanne Reid, Loreena Hill, Lana Dixon, Patrick Donnelly, Paul Slater, Alyson Hill, Susan E Piper, Theresa A McDonagh, Donna Fitzsimons

**Affiliations:** 1 School of Nursing and Midwifery, Queen’s University Belfast, Belfast, UK; 2 Royal Victoria Hospital, Belfast Health and Social Care Trust, Belfast, UK; 3 Ulster Hospital, South Eastern Health and Social Care Trust, Belfast, UK; 4 Institute of Nursing and Health Research, Ulster University, Belfast, UK; 5 Nutrition Innovation Centre for Food and Health, Ulster University, Belfast, UK; 6 Department of Cardiovascular Research, King’s College London, James Black Centre, London, UK; 7 Kings College Hospital NHS Foundation Trust, London, UK

**Keywords:** Cachexia, prevalence, heart failure, sequential phased, quantitative, qualitative, caregiver

## Abstract

**Background:**

Cardiac Cachexia is a wasting syndrome that has a significant impact on patient mortality and quality of life world-wide, although it is poorly understood in clinical practice.

**Aim:**

Identify the prevalence of cardiac cachexia in patients with advanced New York Heart Association (NYHA) functional class and explore its impact on patients and caregivers.

**Design:**

An exploratory cross-sectional study. The sequential approach had two phases, with phase 1 including 200 patients with NYHA III-IV heart failure assessed for characteristics of cardiac cachexia. Phase 2 focussed on semi-structured interviews with eight cachectic patients and five caregivers to ascertain the impact of the syndrome.

**Setting/participants:**

Two healthcare trusts within the United Kingdom.

**Results:**

Cardiac Cachexia was identified in 30 out of 200 participants, giving a prevalence rate of 15%. People with cachexia had a significantly reduced average weight and anthropometric measures (*p* < 0.05). Furthermore, individuals with cachexia experienced significantly more fatigue, had greater issues with diet and appetite, reduced physical wellbeing and overall reduced quality of life. C-reactive protein was significantly increased, whilst albumin and red blood cell count were significantly decreased in the cachectic group (*p* < 0.05). From qualitative data, four key themes were identified: (1) ‘Changed relationship with food and eating’, (2) ‘Not me in the mirror’, (3) ‘Lack of understanding regarding cachexia’ and (4) ‘Uncertainty regarding the future’.

**Conclusions:**

Cardiac cachexia has a debilitating effect on patients and caregivers. Future work should focus on establishing a specific definition and clinical pathway to enhance patient and caregiver support.

**What is already known about the topic?**

- Cardiac cachexia is a debilitating wasting syndrome which frequently is not assessed in clinical practice.
- Much of the research effort to date has focussed on cancer cachexia and, as such, the impact of cardiac cachexia on patients and caregivers remains poorly understood.

**What this paper adds?**

- A prevalence rate of 15%, shows that this syndrome is relatively common within the advanced NYHA functional class.
- A description of challenges in identifying the syndrome and potential priorities for current clinical practice.
- Novel qualitative findings, portraying the severe impact of the syndrome on the daily lives of patients and caregivers – as well as their lack of understanding of cardiac cachexia.

**Implications for practice theory or policy**

- Comprehensive assessment of the syndrome is crucial to its management – clinicians need to be more aware of cardiac cachexia.
- Further work should focus on developing a definition specific to cardiac cachexia, to aid this identification.
- Patients and caregivers need to be better informed about the syndrome, its associated prognosis, and management strategies.

## Introduction

Cachexia is popularly understood as a ‘bad sign’, presenting clinically as a complex and multifactorial wasting syndrome, which frequently goes unrecognised in clinical practice. It is typically associated with significant and unintentional rapid weight loss, a reduction in skeletal muscle mass and reduced quality of life.^1^ The syndrome has a global impact, affecting nine million people worldwide.^2^ Cachexia presents in patients with a chronic illness, such as cancer,^3^ chronic obstructive pulmonary disorder,^4^ renal disease^5,6^ and heart failure.^7^ The focus of the present study is cardiac cachexia within a heart failure population with advanced NYHA functional class. In 2014, it was estimated that 1.2 million individuals were suffering from cardiac cachexia in Europe, with a 1-year estimated mortality rate of 20%–40%.^1^ This rate is not surprising, as it is well established that malnutrition in heart failure is associated with increased mortality; with individuals with cachexia having a 50% mortality rate at 18 months follow up.^8^

**Figure 1.**
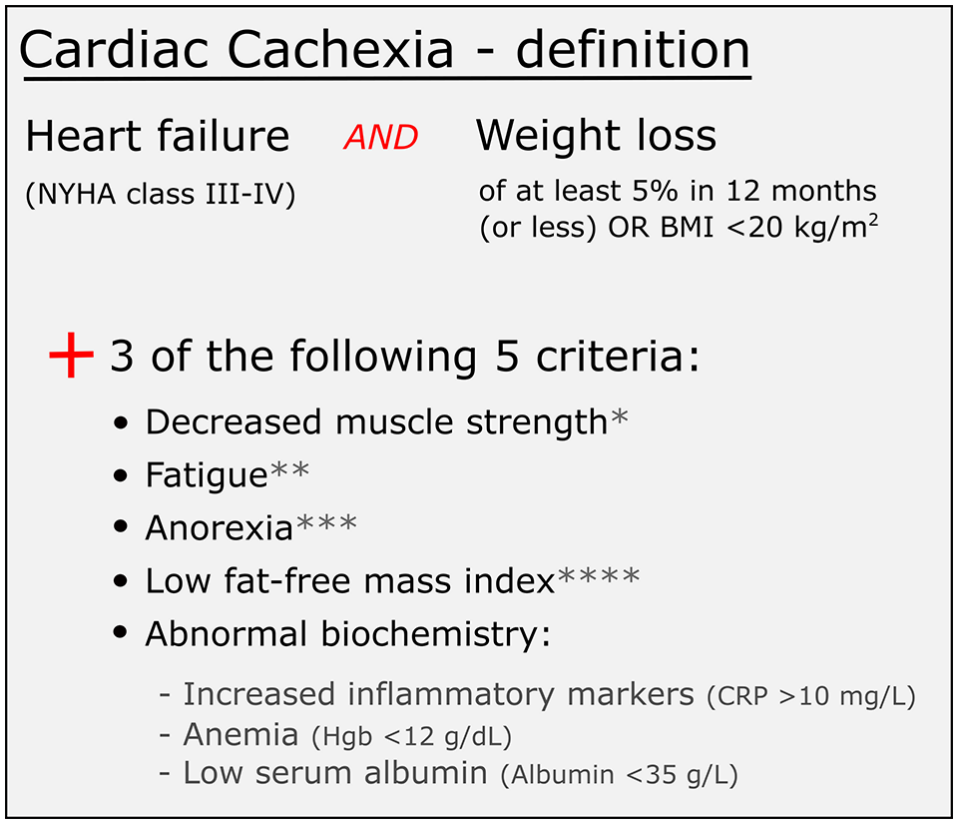
Diagnostic criteria for cachexia, adapted from Evans et al.^13^ CRP: C-reactive protein; Hgb: haemoglobin. *Lowest tertile.^17^ **Physical or mental weariness resulting from exertion; unable to continue exercise at the same intensity without a decrease in performance.^18^ ***Limited food intake (total intake of calories is less than 20 kcal/kg body weight/d; <70% usual food intake).^19^ ****Depletion of lean tissue (i.e. mid upper arm circumference <10th percentile for age and gender).^20^

Elevated levels of pro-inflammatory cytokines are thought to be an important factor in the pathogenesis of cardiac cachexia and other types of cachexia such as cancer, though the cytokine profile varies between each syndrome.^9^ Much of the research in cachexia has focussed on cancer cachexia, with work now progressing to allow earlier identification of the syndrome – ‘pre-cachexia’.^10^ Conversely, cardiac cachexia has received relatively little research effort and remains poorly recognised in clinical practice.^11^ One barrier to effective diagnosis and treatment is the lack of a definition and biomarker specific to cardiac cachexia.^12^ As such, this study uses a commonly accepted consensus definition^13^ relevant to all types of cachexia, which states that cachexia is present when a patient has weight loss of at least 5% in ⩽12 months or body mass index <20 kg/m^2^, plus three of five other criteria (see Figure 1). This definition has been used in other studies investigating cardiac cachexia and is not specific only to a heart failure population.^14–16^

Despite this, studies detailing the prevalence of cardiac cachexia have increased in recent years,^21^ with prevalence rates ranging from 10% to 39%.^8,15,16,22–33^ Particularly within the United Kingdom, data is limited, with only two studies determining prevalence,^8,23^ the last conducted in 2003 and using an outdated indicator of cachexia (weight loss >6%).^23^ In addition, the impact of cardiac cachexia on the daily lives of patients remains poorly understood. There is a dearth of qualitative data, with only one study to date detailing the experiences of food and food intake in heart failure patients,^34^ whilst no studies have included caregivers.

Research focussing on the prevalence and effects of cardiac cachexia would allow better understanding of the syndrome, enhancing current treatment strategies and highlighting priorities for patient care. This sequential, phased, cross-sectional study aims to address the current gaps in our collective knowledge, identifying cachexia and detailing its impact on patients and caregivers with novel qualitative findings.

## Methods

### Design

Use of a cross-sectional sequential phased study design was appropriate, given the dearth of both quantitative and qualitative research in this field. Furthermore, pairing anthropometric and self-report measures with semi-structured interviews (both patients and caregivers) allows for a holistic understanding of the impact of the syndrome. On the day of patient recruitment, quantitative data was collected and analysed to determine if individuals had cachexia or not **(phase 1)**. Within 2 weeks of recruitment, participants were invited to interview, and qualitative data was subsequently transcribed and analysed **(phase 2)**.

### Phase 1

#### Population

A sample size calculation, based on a 5% margin of error, a 95% confidence level and a response distribution of 50%, gave a necessary sample size of 362 patients. However, due to difficulties recruiting as a result of COVID-19 restrictions (see limitations section for further detail) only 204 NYHA class III and IV heart failure patients were recruited. This gave 85% statistical power and in total four participants were excluded from the dataset – one due to surgery and three due to non-cachectic weight loss.

#### Setting

Recruitment took place at heart failure clinics and inpatient wards at four UK hospitals (between July 2019 and May 2021).

#### Recruitment

Two weeks prior to the commencement of patient recruitment, posters referring to the study were placed in reception and waiting areas frequented by patients. Heart failure nurses or a cardiologist helped to identify patients who met the inclusion and exclusion criteria (see Table 1). Only NYHA III and IV heart failure patients were included, as cachexia is associated with increased functional class^35^ (see inclusion/exclusion criteria in Table 1). In addition to their functional class, healthcare professionals also made a judgement on whether patients were mentally and physically capable of participation, before outlining the study to them. Interested patients were then directed to the researcher (MAC) who explained the study further and provided an invitation pack. If content, patients provided written informed consent and then completed data collection.

**Table 1.** Patient and caregiver inclusion and exclusion criteria.

Patient inclusion criteria – phase 1	Patient exclusion criteria – phase 1
Aged 18 and over	<18 years
Able to read, write and speak English	NYHA class I–II
NYHA class III–IV	
Physically and mentally capable of participation	
Willing to be involved	
Patient inclusion criteria – phase 2	Exclusion criteria – phase 2
Identified as having cardiac cachexia in phase 1, as per the Evans et al.^13^ consensus definition criteria (see Figure 1)	Not identified as having cardiac cachexia
Physically and mentally capable of participation*	
Willing to be involved	
Caregiver inclusion criteria – phase 2	Caregiver exclusion criteria – phase 2
Caregiver to patient participant in phase 2	Contact with patient <20 h per week
Contact with patient >20 h per week	
Physically and mentally capable of participation*	
Willing to be involved	

* Whether patients and caregivers were physically and mentally capable of participating in the study or not was determined by advanced heart failure nurses or consultant cardiologists, who helped identify eligible participants.

#### Anthropometric measurements

##### Mid-upper arm circumference and skinfold thickness

Mid upper arm circumference and triceps skinfold thickness were measured in triplicate on the dominant arm, using a standard protocol.^36,37^ Mid upper arm circumference is suggested as an indicator of lean tissue depletion in the consensus definition of cachexia^13^ and was used in the current study, whilst skinfold thickness is also useful in detecting changes in body composition in combination with body mass index and other measures.^25^ A Holtain Tanner/Whitehouse skinfold caliper (Holtan LTD. Crymych, UK) and disposable tape measure were used. Results were compared to normative values,^38^ with the fifth percentile chosen as an appropriate cut-off for a ‘low scoring’ result.^39^

##### Muscle strength

Hand Grip Strength, a strong indicator of morbidity and mortality,^13^ was measured on both arms using a Baseline hydraulic hand dynamometer (Fabrication enterprises inc. White Plains, NY 10602 U.S.A.). A standard protocol^40^ was used – seated position with elbow at 90°, allowing three attempts.

##### Other calculated measures

Using the previously described anthropometric measures, several other measures were calculated and compared to normative values^38^: mid upper arm muscle circumference [cm] = mid arm circumference (cm) – 0.314 × triceps skinfold thickness (mm), upper arm area = mid upper arm circumference^2^ ÷ 12.56, upper arm muscle area = mid upper arm muscle circumference^2^ ÷ 12.56, upper arm fat area = upper arm area – upper arm muscle area.^38^

#### Self-report instruments

As outlined previously,^41^ patients completed three validated instruments/scales: (1) EuroQol 5 Dimension 5 Level, (2) Functional Assessment of Chronic Illness Therapy-fatigue and (3) Functional Assessment of Anorexia/Cachexia Therapy.

#### Collection of relevant patient data and biochemistry

In addition to anthropometric measurements and self-report data, patients’ medical records were also reviewed for information relevant to the present study, such as weight, prescribed medication, existing co-morbidities and recent biochemistry results.

#### Analysis

Quantitative data were entered into SPSS version 26 (IBM Corp., Armonk, New York, USA) and analysed. Firstly, participants were split into those who did (cachectic) or did not (not cachectic) have cachexia using Evans et al.^13^ criteria (see Figure 1 for criteria and appendix table 1 for a summary of how many individuals met the criteria in each group). Subsequently, analysis of the data was based on the measurement type and, where relevant, its normality of distribution as determined by Shapiro-Wilk test. The appropriate statistical tests were applied, including *t*-test (normally distributed), Mann-Whitney *U* (non-normal) and chi squared (categorical data). Statistical significance was given as *p* < 0.05.

### Phase 2

#### Population

Phase 1 data were analysed in relation to the Evans et al.^13^ consensus definition of cachexia (see Figure 1) to determine who did or did not have cachexia. Only willing patients identified as suffering from the syndrome were included in phase 2. Eligible patients (those identified as having cachexia and willing to participate) were invited to interview.

#### Setting

Four of the 13 interviews were conducted by phone (instead of face to face), due to COVID-19 restrictions. Remaining interviews were conducted in the homes of patients and caregivers.

#### Recruitment

When consenting for phase 1, patients were asked if they would consider participation in a potential interview (Phase 2). Following identification of the cachectic sub-population, interested patients were invited to participate in Phase 2 over the telephone. Patients were also asked to nominate a caregiver to participate in a separate interview. An information pack was posted to participants and after a 1 week cool-off period they were asked if they would still like to participate. If willing, a time for a face to face or telephone interview was then arranged.

#### Interview and analysis

One researcher (MAC) conducted a semi-structured interview with each patient, with questions following a laddered style approach. Interviews were digitally recorded and then transcribed verbatim. Interviews lasted an average of 43 (15–64) min. Data were analysed by thematic analysis (MAC), using the six step approach of Braun and Clarke.^42^ Themes were developed and refined by several members of the research team (MAC, LH, JR, DF, SEP, TAMcD), to ensure rigour.^43^

## Research ethics and approvals

Ethical approval was granted by the Office for Research Ethics Committees Northern Ireland (REC reference: 23/NI/0092), whilst local governance approval was granted by the participating recruitment sites. Written informed consent was obtained from all participants before participation.

## Results

### Phase 1: Prevalence and impact

Of the 200 patients recruited into this study, cachexia was identified in 30 individuals using the criteria of Evans et al.,^13^ giving a prevalence rate of 15%. The overall population was predominantly male (65.5%) (see Table 2 for general characteristics), with an average age of 74.4 years. The entire population suffered from a range of comorbidities (commonly atrial fibrillation, hypertension and chronic kidney disease), with an average Charlson Comorbidity Index value of 5.9. There was no significant difference in the prevalence of comorbidities between the groups, with the exception of cancer (23.3% vs 8.2% in the not cachectic group). Cancer patients were included in this analysis to ensure the study population was representative of the general heart failure population. However, the influence of cancer patients on findings was minimal – as trends remained similar when these patients were excluded, whilst a sensitivity analysis showed a minimal impact (value of 91.8%) of cancer diagnosis on determination of cachexic status. The percentage of NYHA class IV patients was significantly greater in the cachectic group, whilst the class III percentage was significantly reduced. In terms of medications, aldosterone antagonist use was significantly reduced in the cachectic group.

**Table 2.** General characteristics.

Descriptor	All (*n* = 200)	Not cachectic (*n* = 170)	Cachectic (*n* = 30)	Significance
Age (years), mean ± SD	74.4 ± 12.9	74.2 ± 13.1	75.6 ± 11.7	NS
Sex (male), *n* (%)	131 (65.5)	112 (65.9)	19 (63.3)	NS
CCI score, mean ± SD	5.9 ± 2.1	5.8 ± 2.12	6.1 ± 1.7	NS
Ischaemic aetiology, *n* (%)	74 (37)	62 (36.5)	12 (40)	NS
Hypertension, *n* (%)	85 (42.5)	71 (41.8)	14 (46.7)	NS
Previous MI, *n* (%)	33 (16.5)	29 (17.1)	3 (13.3)	NS
Atrial fibrillation, *n* (%)	119 (59.5)	99 (58.8)	18 (63.3)	NS
Implanted device, *n* (%)	55 (27.5)	44 (25.9)	11 (36.7)	NS
CRT-P, CRT-D, *n* (%)	19 (9.5)	14 (8.8)	3 (13.3)	NS
ICD, *n* (%)	23 (11.5)	20 (11.8)	3 (10)	NS
Pacemaker, *n* (%)	13 (6.5)	9 (5.3)	3 (13.3)	NS
Cancer,* *n* (%)	21 (10.5)	13 (8.2)	6 (23.3)	0.01
Diabetes, *n* (%)	68 (34)	58 (34.7)	9 (30)	NS
Chronic Kidney Disease,** *n* (%)	72 (36)	63 (37.1)	9 (30)	NS
COPD,*** *n* (%)	40 (20)	31 (18.8)	8 (26.7)	NS
HFrEF, *n* (%)	94 (47)	80 (47.1)	13 (46.4)	NS
HFmrEF, *n* (%)	56 (28.4)	48 (28.4)	8 (28.6)	NS
HFpEF, *n* (%)	49 (24.6)	41 (24.5)	7 (25)	NS
NYHA class III, *n* (%)	189 (94.5)	165 (97.6)	23 (76.7)	0.01
NYHA class IV, *n* (%)	11 (5.5)	4 (2.4)	6 (23.3)	0.01
Oedema present, *n* (%)	121 (60.5)	105 (61.8)	15 (53.3)	NS
Medication use				
Digoxin, *n* (%)	34 (17)	27 (15.9)	6 (23.3)	NS
Sacubitril/valsartan, *n* (%)	28 (14)	27 (15.9)	1 (3.3)	NS
ACE/ARB, *n* (%)	90 (45)	70 (41.2)	20 (66.7)	NS
Beta blockers, *n* (%)	152 (76)	128 (75.3)	24 (80)	NS
Metolazone, *n* (%)	21 (10.5)	18 (10.6)	3 (10)	NS
Loop diuretics, *n* (%)	159 (79.5)	134 (79.4)	24 (80)	NS
Aldosterone antagonists, *n* (%)	103 (51.5)	91 (54.1)	11 (36.7)	0.03
Daily furosemide dose (mg)	51	52	45	NS

CRT-P: cardiac resynchronisation therapy pacemaker; CRT-D: cardiac resynchronisation therapy defibrillator; ICD: implantable cardioverter-defibrillator; COPD: Chronic Obstructive Pulmonary Disorder; HFrEF: heart failure with reduced ejection fraction; HFmrEF: heart failure with mid-range ejection fraction; HFpEF: heart failure with preserved ejection fraction; NYHA: New York Heart Association. For this study a HFrEF = an ejection fraction <40%, HFmrEF = 40%–49% and HFpEF = ⩾50%.

Detail on the measure and units being used are included after each descriptor/outcome (typically mean and standard deviation). Statistical significance (*p* < 0.05) was determined by comparing the not cachectic and cachectic groups using a Mann-Whitney *U* test.

* Cancer was confirmed if patient was diagnosed in last 5 years and/or on active treatment, or receiving cancer related palliative care. ** CKD was confirmed where eGFR of <35 mL/min. *** COPD was confirmed where FEV < 50% predicted.

The cachectic group had significantly reduced weight compared to the not cachectic group (61.4 vs 86.7 kg), and a significantly lower body mass index of 21.8 (see Table 3). The non-oedematous weight loss over 1 year for the cachectic group was 7.1 kg, significantly greater than the not cachectic group which averaged 1.1 kg. Compared to those without cachexia, the cachectic group showed a significant reduction in all anthropometric measures such as mid upper arm circumference and triceps skinfold thickness (see Table 3). These reductions were pronounced, with the percentage difference between the groups ranging from 16.2% to 41.5%.

**Table 3.** Anthropometric values and self-report outcomes (questionnaires).

Outcome measure	All (*n* = 200)	Not cachectic (*n* = 170)	Cachectic (*n* = 30)	Significance
Weight (kg)	82.8 ± 24.9	86.7 ± 24.5	61.4 ± 13.9	<0.01
BMI	28.6 ± 7.6	29.9 ± 7.4	21.8 ± 4.4	<0.01
Non-oedematous weight loss 1 year (kg)	2.0 ± 3.6	1.1 ± 2.3	7.1 ± 5.4	<0.01
Mid upper arm circumference (cm)	29.9 ± 5.2	30.8 ± 4.9	25.1 ± 3.7	<0.01
Skinfold thickness (mm)	15.5 ± 6.7	16.2 ± 6.67	11.5 ± 5.2	<0.01
Mid upper arm muscle circumference (mm)	250.9 ± 37.6	257.1 ± 35.5	215.4 ± 28.6	<0.01
Upper arm area (mm^2^)	73.5 ± 25.8	77.4 ± 25.3	51.4 ± 15.5	<0.01
Upper arm muscle area (cm^2^)	51.2 ± 15.5	53.6 ± 15.1	37.6 ± 10.1	<0.01
Upper arm fat area (cm^2^)	22.1 ± 12.5	23.6 ± 12.7	13.8 ± 7.5	<0.01
Grip strength right (kg)	16.2 ± 10.9	17.1 ± 11.2	11.4 ± 7.2	0.01
Grip strength left (kg)	15.1 ± 10.5	16 ± 10.9	10 ± 6.3	<0.01
FACIT Fatigue score	23.1 ± 12.3	24.0 ± 12.3	17.8 ± 10.7	<0.01
FAACT – Physical wellbeing	18.1 ± 6.1	18.5 ± 5.8	15.6 ± 6.9	0.03
FAACT – Social wellbeing	22.9 ± 5.5	22.9 ± 5.6	22.6 ± 4.8	NS
FAACT – Emotional wellbeing	16.5 ± 5.7	16.7 ± 5.7	15.9 ± 6.1	NS
FAACT – Functional wellbeing	14.6 ± 6.6	14.8 ± 6.7	14.0 ± 6.1	NS
FAACT – Anorexia and cachexia subscale	34.5 ± 8.6	35.9 ± 7.2	26.6 ± 11.6	<0.01
FAACT – Total score	107.0 ± 22.5	109.1 ± 21.7	94.9 ± 23.5	0.04
EQ – Mobility	3.1 ± 1.1	3.0 ± 1.1	3.5 ± 0.9	0.02
EQ – Self-care	2.0 ± 1.1	1.9 ± 1.1	2.1 ± 1.1	NS
EQ – Usual activities	3.2 ± 1.2	3.1 ± 1.2	3.7 ± 1.1	<0.01
EQ – Pain/discomfort	2.5 ± 1.2	2.4 ± 1.2	2.5 ± 1.5	NS
EQ – Anxiety/depression	2.0 ± 1.1	1.9 ± 1.1	2.2 ± 1.2	NS
EQ – Index value	0.6 ± 0.3	0.6 ± 0.2	0.4 ± 0.5	0.024
EQ – Visual analogue scale	53.1 ± 20.3	54.1 ± 19.8	47.6 ± 22.5	NS

BMI: Body Mass Index; EQ: EuroQol 5 Dimension 5 Level; FACIT: Functional Assessment of Chronic Illness Therapy; FAACT: Functional Assessment of Anorexia/Cachexia Therapy.

All values are reported as the mean, plus and minus the standard deviation. Statistical significance (*p* < 0.05) was determined by comparing the not cachectic and cachectic groups using a Mann-Whitney *U* test (except for mid upper arm muscle circumference for which an independent samples *t*-test was used).

Results for self-report instruments are shown in Table 3. In terms of quality of life, patients with cachexia reported significantly greater fatigue, based on responses to the Functional Assessment of Chronic Illness Therapy-fatigue scale. According to results from the Functional Assessment of Anorexia/Cachexia Therapy scale, patients with cachexia had significantly reduced physical wellbeing, greater issues with diet and appetite – as per the anorexia cachexia subscale – and a worse total score across the instrument. Conversely, the social, emotional and functional subscales showed no differences between the groups (*p* > 0.05). For the EuroQol 5 Dimension 5 Level, the cachectic group had significantly greater issues with their mobility and were experiencing greater changes to their usual activities, compared to the not cachectic group. The self-care, pain/discomfort and anxiety/depression subscales showed no significant difference between the groups. However, the cachectic group had significantly reduced quality of life overall – as per the reported index value.

Regarding biochemistry and haematology (see Table 4), patients with cachexia had significantly increased c-reactive protein levels (average of 30.7 vs 15.3 mg/L in the not cachectic group), and significantly decreased albumin (37.9 vs 40.2) and red blood cell count (3.8 vs 4.2). Remaining blood measures showed variations between the groups, but none of these were statistically significant. Average haemoglobin was decreased in the cachectic group, whilst creatinine, platelet count, alkaline phosphatase, gamma-glutamyl transferase and brain natriuretic peptide all showed non-significant increases.

**Table 4.** Biochemistry results.

Blood measure	All (*n* = 200)		Not cachectic (*n* = 170)		Cachectic (*n* = 30)		Significance
	Sample size (*n*)	Mean ± SD	Sample size (*n*)	Mean ± SD	Sample size (*n*)	Mean ± SD	
CRP (mg/L)	143	17.9 ± 23.1	119	15.3 ± 19.4	24	30.7 ± 34.3	0.04
Albumin (g/L)	130	39.8 ± 5.2	107	40.2 ± 5.2	22	37.9 ± 5	<0.05
Creatinine (µmol/L)	189	134 ± 60.6	156	132.4 ± 51.8	27	140.4 ± 98.7	NS
Platelet count	148	232.2 ± 88.8	125	228.5 ± 83.6	23	252.4 ± 113.1	NS
Haemoglobin (g/L)	162	116.6 ± 25.1	136	114.5 ± 26.9	26	111.9 ± 11.8	NS
Red blood cell count	128	4.1 ± 0.7	105	4.2 ± 0.8	23	3.8 ± 0.6	0.03
ALP (U/L)	125	106.4 ± 43.3	104	105 ± 43	20	109.3 ± 42.7	NS
GGT (U/L)	145	94.7 ± 113	120	88.7 ± 100.4	24	119.7 ± 162.1	NS
BNP (ng/L)	135	6316.5 ± 6627.2	112	5866.5 ± 5973.5	22	8592.14 ± 9226.6	NS

CRP: C-reactive protein; ALP: alkaline phosphatase; GGT: Gamma-glutamyl transferase; BNP: brain natriuretic peptide.

All values are reported as the mean, plus and minus the standard deviation. Statistical significance (*p* < 0.05) was determined by comparing the not cachectic and cachectic groups using a Mann-Whitney *U* test (except for red blood cell count for which an independent samples *t*-test was used).

From the perspective of proportions, a significantly greater proportion of patients in the cachectic group displayed criteria from the consensus definition of cachexia^13^ when compared to the not cachectic group, except for fatigue (see Supplemental Appendix Table 1). Decreased muscle strength was most common (80% of patients with cachexia), followed by low fat-free mass index (76.7%) and abnormal biochemistry (74.1%). Comparatively, the not cachectic group commonly presented decreased muscle strength (56% of the not cachectic patients), followed by fatigue (47.5%) and abnormal biochemistry (38.7%).

### Phase 2: Patient and caregiver experiences

Semi-structured interviews with patients with cachexia and their caregivers (see Table 5) highlighted four key themes associated with the syndrome: (1) Changed relationship with food and eating, (2) Not me in the mirror, (3) Lack of understanding regarding cachexia and (4) Uncertainty regarding the future.

**Table 5.** Patient details for semi-structured interviews.

Identifier	Age	Gender	NYHA class	CCI value	Caregiver also interviewed?	Caregiver gender	Caregiver relationship	Interview location
Patient 1	72	M	NYHA 4	7	Y – Caregiver 1	F	Wife	Home
Patient 2	76	M	NYHA 3	9	Y – Caregiver 2	F	Wife	Home
Patient 3	61	M	NYHA 4	4	N	-	-	Home
Patient 4	46	F	NYHA 4	4	Y – Caregiver 3	M	Partner	Telephone
Patient 5	71	F	NYHA 3	5	N	-	-	Home
Patient 6	85	M	NYHA 3	5	Y – Caregiver 4	F	Wife	Home
Patient 7	88	F	NYHA 3	6	N	-	-	Telephone
Patient 8	77	M	NYHA 4	8	Y – Caregiver 5	F	Wife	Telephone

NYHA: New York Heart Association; CCI: Charleston Co-Morbidity Index.

### Changed relationship with food and eating

Patients with cachexia highlighted a change in their relationship with food and eating, referring to eating as something they now ‘make’ themselves do without enjoyment, often just to placate their caregiver.

*‘Yes, I would be a picky eater. If my wife cooks something I don’t like to say, ‘oh I don’t feel like that’. I would force myself to eat things’.* (Patient 8)

Caregivers noted this change in habit and were concerned about adequate nutrition being provided due to the patient’s lack of interest in food.

*‘It’s worrying for me because I don’t know if he’s getting the right things’.* (Caregiver 5)

Caregivers did not understand the change and focussed on trying to ensure adequate nutrition, though often felt frustrated with their lack of progress.

*‘. . ..there is nothing really I can do about it [reduced appetite] and nothing he can do about the way that he feels. But sometimes I feel like I just want to get him and shake him and that makes me feel bad. . .’..* (Caregiver 1)

The importance of food to social interactions is apparent, as is the clear pressure that cachexia places on family dynamics.

### Not me in the mirror

Patients typically had a changed and negative perception of themselves, which was linked to their recent weight loss – commenting ‘that’s not me in that mirror’.

*‘Yes. The weight has fell off me. I am like something out of a [concentration] camp. I am very thin’.* (Patient 5)

Caregivers were similarly aware of the physical changes in their loved ones and emotionally impacted by this.

*‘He had a beautifully tailored dress suit. He was going to a dinner and he put it on and that is when I could have cried. It was swinging’.* (Caregiver 4)

It was evident that both patients and caregivers found this unintentional weight loss distressing.

### Lack of understanding regarding cachexia

The weight loss and physical changes experienced by patients evidently was a source of concern, though there was the perception that healthcare professionals did not share in this. Instead, patients felt as though healthcare professionals were just ‘*fobbing you off’*.

*‘You went to the doctors, and they go to great length and the hospital weighing you but that is the end of that. Nobody says well we need to investigate this or that’.* (Patient 7)

Caregivers shared the patient’s confusion and appeared to want information to assist them in understanding the cause of the weight loss.

*‘I don’t understand where the weight loss is coming from. . .. haven’t got cancer. I just don’t understand why’. . .* (Caregiver 2)

Due to their lack of understanding and poor information provision, patients and caregivers falsely attributed weight loss to a variety of causes, including cancer, medications, hyperthyroidism, and diabetes.

*‘[Regarding weight loss] I am on a lot of drugs too. Once a year I get this drug for osteoporosis. You only get it for three years, so I don’t know if that has done it too’.* (Patient 5)

Even though weight loss was noted and distressing to patients and caregivers, there was little clinical recognition of it, nor any advice or support from the clinical team regarding management.

### Uncertainty regarding the future

Overall, patients and caregivers recognised the holistic impact cachexia had as a warning sign.

*‘He is not the person he was six months ago, definitely not; even in his demeanour, his attitude. It is sad, it is heart-breaking. . .’* (Caregiver 1)

Many patients expressed fears for the future.

*‘. . .resigned myself to the fact that I am never going to get any better. The only thing I have got to look forward to is Milltown [cemetery]’.* (Patient 2)

Given the previously noted concerns that patients and families held regarding cachexia, it is unsurprising that many were worried about their health and prognosis.

## Discussion

### Main findings

This sequential phased study provides an updated prevalence of cardiac cachexia and a unique insight into patients’ and caregivers’ experience of the syndrome. Findings clarify the debilitating impact of the syndrome, through physical effects such as weight and muscle loss which contribute to fatigue, reduced quality of life, a change in how the patient perceives themselves and worries for the future. Of concern from this novel qualitative data, is that patients and caregivers have a poor understanding of cachexia, highlighting this population’s need for further support.

### What this study adds

The 15% prevalence rate found in the sample population indicates cardiac cachexia is a relatively common syndrome within the advanced heart failure population, similar to more dated studies which reported prevalence data^8,15,16,22–33^ ranging from 10% to 39%.

Weight loss is arguably the most important clinical indicator of cachexia and a predictor of mortality^44^ but within a heart failure cohort, this factor is challenging to identify, as oedema (present in 60.5% of patients in the present study) can mask weight loss and muscle wasting.^45^ In addition, a reduction in fluid retention due to diuretics can be confused with reduction in body mass index, although this was accounted for in the present study. Another issue complicating identification of cardiac cachexia, is the tendency for heart failure patients to have an elevated body mass index compared to the general population.^46^ From baseline results, 85% of participants in this study had an average body mass index of 29.9 kg/m^2^, meaning they were on the borderline of being classed as obese. The body mass index cut-off of <20 kg/m^2^ from the consensus definition of cachexia^13^ was met by 10.5% of participants, indicating a higher cut-off value may be appropriate in this population.

As highlighted in Table 3, cachectic patients exhibited greater loss of fat than muscle mass – though both were substantial. Similar findings have been reported in related work,^15,16,25^ whereby fat reserves acts as a protection from more accelerated muscle loss, or fat loss may precede lean tissue loss.^16,47^ Such decreases in anthropometric measures may aid in recognition of the syndrome, particularly mid upper arm skinfold thickness, which is not invasive, easy to obtain and showed a clear difference between groups. The impact of physical changes on both the patient and caregiver was highlighted in the qualitative theme ‘not me in the mirror’, demonstrating they were continually aware of physical changes and perceived these negatively. This visual phenomena of cachexia has a multifaceted psychosocial impact, and for many patients is interpreted as a bad sign.^6^

Within this study cachectic patients experienced greater fatigue, which is likely related to malnutrition, loss of fat, muscle tissue, decreased muscle strength and reduced red blood cell count. Interestingly, cachectic individuals had reduced physical wellbeing on the Functional Assessment of Anorexia/Cachexia Therapy scale, but no change in other quality of life measures such as social or emotional wellbeing, when compared to the not cachectic group. Similar non-significant differences have been reported previously^48^ and may indicate that cachectic patients have sufficient support mechanisms at home. The anorexia cachexia subscale showed the largest difference between groups, with cachectic patients reporting reduced appetite and food intake. Such findings are not uncommon,^15,30,34^ but serve to highlight the dietary issues experienced by this population.

Data from the Functional Assessment of Anorexia/Cachexia Therapy scale shows cachectic patients frequently reported reaching satiety quickly, having difficulty with rich or heavy food, losing interest in food and getting pressure from family/friends to eat. Interestingly, being worried about their weight was one of the more infrequently reported issues, which seems to support the aforementioned challenges to weight determination in this population – as well as a lack of knowledge regarding cachexia. Qualitative data replicated and expanded upon these answers, describing individuals who no longer had a positive relationship with food and who now only ate out of necessity and a desire to placate their caregiver. Similarly, patients and caregivers did not understand the significance of the weight loss or know anything about cardiac cachexia. There was a sense of frustration in these individuals and a desire to understand and potentially better manage this worrying symptom. This finding is not novel, with other work detailing the need for better information provision from healthcare professionals.^6,34^ Enhanced information provision would help to alleviate the concerns for both patient and caregivers, improving quality of life during their remaining time.

Qualitative findings provided insight into the concern patients and caregivers have regarding the future. Many had contemplated the consequences of further decline in their condition and the possibility of death – often in third person, indicating a fear that their concerns would become real. Input from caregivers demonstrated they also are greatly impacted by their loved one’s decline, feeling worried about ensuring adequate nutrition and what the future holds.

### Strengths and weaknesses of the study

This study provides an updated prevalence rate for cardiac cachexia and novel insight into the impact of this syndrome, though there are limitations to be considered. Despite best efforts to recruit all class III and IV patients regardless of their diagnosis, there is potential for referral bias, which could have impacted the representativeness of the population and the calculated prevalence rate of cachexia. A greater sample size would have improved the quality and reliability of results for both phases of work, though data collection was significantly impacted by COVID-19 restrictions and therefore phase 1 stopped before reaching the intended sample size.^41^ Despite this, 85% is generally considered adequate for studies of this kind.^49^ COVID-19 restrictions also impacted recruitment for phase 2, meaning data saturation was not reached. However, there was evidence for repetition of key themes before data collection ceased. Finally, it should be noted that sarcopenia is also common in the older heart failure patient, along with cachexia and frailty,^50^ and there is therefore some possibility of misdiagnosis between these conditions. Whilst difficult to control for, such crossover should have been fairly minimal whilst using criteria from the consensus definition of cachexia^13^ – as sarcopenia is primarily associated with age rather than chronic conditions, does not always result in weight loss and is not linked to loss of fat mass (only skeletal muscle mass).

## Conclusion

Cardiac cachexia, an under recognised health concern, was found to affect 15% of the population with advanced NYHA functional class and was associated with significant physical changes and decreased quality of life. Future work should aim to improve identification of the syndrome, through the development of a specific definition for cardiac cachexia, which should involve exploratory biomarker work. Furthermore, this paper highlights the importance of acknowledging the detrimental effects of cardiac cachexia, and developing better strategies to manage the syndrome and to inform and support the patients and family caregivers that it impacts, are urgently needed.

## Supplemental Material

sj-pdf-1-pmj-10.1177_02692163221101748 – Supplemental material for Exploring the prevalence, impact and experience of cardiac cachexia in patients with advanced heart failure and their caregivers: A sequential phased study Click here for additional data file. Supplemental material, sj-pdf-1-pmj-10.1177_02692163221101748 for Exploring the prevalence, impact and experience of cardiac cachexia in patients with advanced heart failure and their caregivers: A sequential phased study by Matthew A Carson, Joanne Reid, Loreena Hill, Lana Dixon, Patrick Donnelly, Paul Slater, Alyson Hill, Susan E Piper, Theresa A McDonagh and Donna Fitzsimons in Palliative Medicine
