# Supplementary material for: Exploring the prevalence, impact and experience of cardiac cachexia in patients with advanced heart failure and their caregivers: A sequential phased study
Source: Palliat Med. 2022 Jun 21;36(7):1118–28. doi: 10.1177/02692163221101748 (PMC9248000; doi:10.1177/02692163221101748)
Supplement: sj-pdf-1-pmj-10.1177_02692163221101748 – Supplemental material for Exploring the prevalence, impact and experience of cardiac cachexia in patients with advanced heart failure and their caregivers: A sequential phased study [file sj-pdf-1-pmj-10.1177_02692163221101748.pdf]

Appendix Table 1. Number of participants displaying indicators of cachexia

*All values are reported as count (n) or percentage (%). Statistical significance (p < 0.05) was determined by comparing the not cachectic and cachectic groups using Chi-Square Test. *Decreased muscle strength = those with an average (from 3 values) right-handed grip strength < the minimum of value of healthy individuals at the same age. **Fatigue = those with a FACIT-Fatigue score < 20. ***Reduced/issues appetite = those with an anorexia and cachexia subscale score < 30. ****Those individuals in the 5th and 10th percentiles for mid-upper arm circumference. *****Abnormal biochemistry = those with elevated (> 10 mg/L) CRP or reduced (< 35 g/L) albumin level or reduced haemoglobin (< 12 g/Dl).*

**Indicator of cachexia**	**All (n = 200)**		**Not cachectic (n = 170)**		**Cachectic (*n*=30)**		**Significance**
	**Sample size (n)**	**n (%)**	**Sample size (n)**	**n (%)**	**Sample size (n)**	**n (%)**	
Decreased muscle strength*	196	117 (59.7)	166	93 (56.0)	30	24 (80.0)	0.01
Fatigue**	200	95 (47.5)	170	76 (44.7)	30	19 (63.3)	NS (0.06)
Reduced/issues appetite***	197	51 (25.9)	168	32 (19.0)	29	19 (65.5)	< 0.01
Low fat-free mas index****	200	66 (33.0)	170	43 (25.3)	30	23 (76.7)	< 0.01
Abnormal biochemistry*****	178	108 (60.7)	150	85 (56.7)	28	23 (82.1)	< 0.01
